# Evidence that modifier loci have no effect on phenotypic severity in SHANK3-linked autism

**Published:** 2014-06

The genetic background in which a disease-associated mutation is expressed can have a profound impact on the resulting disease phenotype. The identification of so-called ‘modifier genes’ can therefore provide insights into disease pathophysiology and could also reveal novel targets for therapeutic intervention. In this study, Joseph Buxbaum and colleagues sought to determine whether modifier loci affect the severity and range of autistic behaviours and developmental deficits associated with the genetic disorder Phelan-McDermid syndrome (PMS). Deletion of *SHANK3*, a gene that has been strongly linked to autism spectrum disorders, is known to be a major cause of the neurobehavioural manifestations of PMS. Buxbaum and co-authors assessed the effects of *Shank3* deficiency in three different strains of laboratory mice. Their extensive behavioural tests demonstrate that phenotypes observed in *Shank3*-deficient mice are largely similar regardless of strain. This indicates that differences in genetic background are unlikely to account for the high variability in disease phenotypes observed in individuals with PMS. This unexpected finding suggests that modifier loci do not play a role in PMS pathogenesis, and other potential contributory factors should be explored to gain a deeper understanding of the underlying disease mechanisms. **Page 667**

